# Diversity matters – heterogeneity of dopaminergic neurons in the ventral mesencephalon and its relation to Parkinson's Disease

**DOI:** 10.1111/jnc.13670

**Published:** 2016-06-27

**Authors:** Daniela Maria Vogt Weisenhorn, Florian Giesert, Wolfgang Wurst

**Affiliations:** ^1^ Helmholtz Zentrum MünchenGerman Research Center for Environmental HealthInstitute of Developmental GeneticsNeuherbergGermany; ^2^ Technische Universität München‐WeihenstephanLehrstuhl für Entwicklungsgenetikc/o Helmholtz Zentrum MünchenNeuherbergGermany; ^3^ Deutsches Zentrum für Neurodegenerative Erkrankungen e. V. (DZNE)Standort MünchenMünchenGermany; ^4^ Munich Cluster for Systems Neurology (SyNergy)Adolf‐Butenandt‐InstitutLudwig‐Maximilians‐Universität MünchenMünchenGermany

**Keywords:** conditional mutagenesis, history, neuroanatomy, Parkinson's disease, single‐cell analysis

## Abstract

Dopaminergic neurons in the ventral mesencephalon (the ventral mesencephalic dopaminergic complex) are known for their role in a multitude of behaviors, including cognition, reward, addiction and voluntary movement. Dysfunctions of these neurons are the underlying cause of various neuropsychiatric disorders, such as depression, addiction and schizophrenia. In addition, Parkinson's disease (PD), which is the second most common degenerative disease in developed countries, is characterized by the degeneration of dopaminergic neurons, leading to the core motor symptoms of the disease. However, only a subset of dopaminergic neurons in the ventral mesencephalon is highly vulnerable to the disease process. Indeed, research over several decades revealed that the neurons in the ventral mesencephalic dopaminergic complex do not form a homogeneous group with respect to anatomy, physiology, function, molecular identity or vulnerability/dysfunction in different diseases. Here, we review how the concept of dopaminergic neuron diversity, assisted by the advent and application of new technologies, evolved and was refined over time and how it shaped our understanding of PD pathogenesis. Understanding this diversity of neurons in the ventral mesencephalic dopaminergic complex at all levels is imperative for the development of new and more selective drugs for both PD and various other neuropsychiatric diseases.

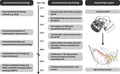

Several decades of research revealed that the neurons in the ventral mesencephalic dopaminergic complex do not form a homogeneous group in respect to anatomy, physiology, function, molecular identity or vulnerability/dysfunction in diseases like Parkinson's disease (PD). Here, we review how this concept evolved and was refined over time and how it shaped our understanding of the pathogenesis of PD. Source of the midbrain image: www.wikimd.org/wiki/index.php/The_Midbrain_or_Mesencephalon; downloaded 28.01.2016. See also Figures 1 and 3 of the paper.

**This article is part of a** special issue on Parkinson disease **.**

**Abbreviations used**
BDNF: brain‐derived neurotrophic factor
CRISPR: clustered regularly interspaced short palindromic repeats
DCC: deleted in colorectal carcinoma
GABA: gamma‐aminobutyric acid
GDNF: glial cell line‐derived neurotrophic factor
IKMC: International Knockout Mouse Consortium
L‐DOPA: L‐3,4‐dihydroxyphenylalanine
MPTP: 1‐methyl‐4‐phenyl‐1,2,3,6‐tetrahydropyridine
PBP: parabrachial pigmented nucleus
PD: Parkinson's disease
SNCD: substantia nigra compacta dorsal tier
SNc: substantia nigra pars compacta
SNCV: substantia nigra compacta ventral tier
SNl: substantia nigra pars lateralis
SNr: substantia nigra pars reticularis
SN: substantia nigra
TH: tyrosine hydroxylase
VTA: ventral tegmental area

One of the most studied neurotransmitter systems in neuroscience is the dopaminergic system. This strong interest is based on the involvement of dopaminergic circuits in a multitude of neurological and psychiatric diseases, including Parkinson's disease (PD), Huntington's disease and both schizophrenia and addiction. The association of dopaminergic neurons with these diseases highlights their role in the control and maintenance of proper motor and cognitive functions, reinforcement, learning and motivational aspects of the healthy brain. The most prominent dopaminergic neurons in the brain are arguably those of the ventral mesencephalon. The death of these neurons is the hallmark of PD and is associated with severe motor dysfunction. Therefore, it is not surprising that the understanding of the function and biology of these neurons is highly important in terms of developing new therapeutic avenues to treat this currently incurable disease. Understanding the function and biology of neurons, however, starts with understanding the details of their neuroanatomy before their molecular characteristics can be considered. Thus, we will first review the neuroanatomy of the dopaminergic system in the brain. This review is written in a historical context to show that it is only through the continuous and parallel development of new technologies that we obtain more insights and a progressively refined understanding of the brain circuitries involved in diseases, for example, PD. Indeed, the discovery and functional characterization of the dopaminergic system is a key example of this process of discovery, in that each technological innovation deepened and advanced our knowledge of this system. These technological advancements first allowed the subdivision of the regions and neurons within the ventral mesencephalon according to their cytoarchitectonic features. This advance was followed by the discovery of the neurochemical identity of the neurons in this region and, subsequently, descriptions of interneuronal connectivity and the location of these neurons in larger circuits. In addition, with the advent of the field of genetics and thus genetic technologies, further in‐depth characterizations were made with respect to the role of dopaminergic neurons in distinct behaviors and their molecular identities. Because of the high interest in the dopaminergic system and its importance in both PD and other neuropsychiatric diseases, a vast amount of literature has already been produced. Thus, we will not be able to acknowledge all of the important work that has been performed by hundreds of investigators in this field. We are therefore forced to select distinct historical and scientific hallmarks; a selection that is, of course, influenced by our own understanding of history and science. Below, we will briefly discuss the discovery and nature of the following four layers of complexity of midbrain (mesencephalic) dopaminergic neurons: cytoarchitectonics, neurochemistry, connectome and molecular identity, as well as the significance of each of these layers to PD.

## Neuroanatomy of the ventral mesencephalic dopaminergic neurons

### Discovery of the substantia nigra and its relationship with Parkinson's disease

**Figure 1:**
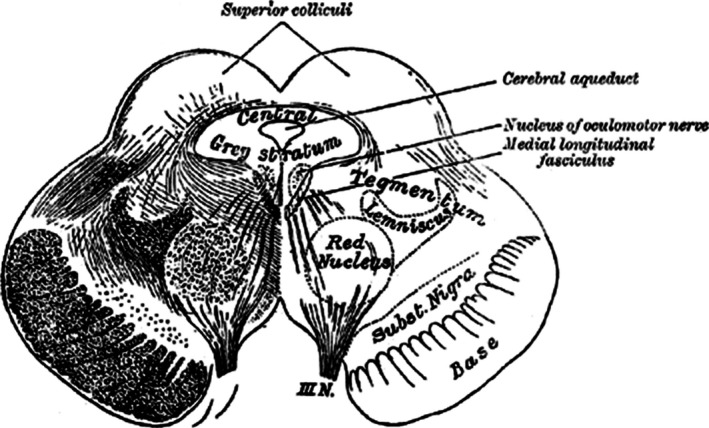
Location of the substantia nigra, ventral to the red nucleus and dorsal to the cerebral peduncles, as depicted in Gray's (1918) anatomy. Source: http://www.wikimd.org/wiki/index.php/The_Mid-brain_or_Mesencephalon; downloaded January 28, 2016.

In 1786, Felix Vicq d'Azyr published the description of a structure termed the ‘locus niger crurum cerebri’ (Vicq d'Azyr 1786). It was later discovered that the dark appearance of this structure, which allowed its recognition without microscopic staining techniques, is because of the exceptionally high content of melanin in its cells. Five years later, Samuel Paul von Soemmering (1791) again described this dark structure; hence, it was also termed the ‘Ganglion of Soemmering’ or substantia nigra Soemmeringi. Thereafter, several neuroscience pioneers, using simple staining methods, such as Nissl staining and staining with gold and potassium chloride, described the substantia nigra (SN) as being located in the midbrain tegmentum. Specifically, the SN was described as being ventral to the red nucleus, between the cerebral peduncles and the medial lemniscus (Burdach 1822; Forel 1877, 1907; Meynert 1884; Sano 1910; Edinger 1911; Gray 1918) (Fig. 1). These pioneers had already discovered three hallmarks of the SN: first, that it is similarly organized in all vertebrates (Luys 1865; Sano 1910; Edinger 1911); second, that the pigmentation of the cells in the SN is obvious in humans (Luys 1865; Forel 1877) and third, that the SN is composed of an upper, cell‐dense layer and a lower, cell‐sparse layer (Mingazzini 1889; Ramon y Cajal 1899–1904, Sano 1910), called the substantia nigra pars compacta (SNc) and the substantia nigra pars reticularis (SNr) respectively. In addition, a third group of cells in the rostral–lateral portion of the SN were cytoarchitectonically demarcated and termed the substantia nigra pars lateralis (Huber *et al*. 1943; Ma 1989).

The history of the discovery of the substantia nigra in the ventral mesencephalon is connected with the discovery of the neuropathological symptoms of PD (reviewed in Parent and Parent 2010). Still, the symptoms of PD, specifically tremor, have been known since ancient times. These symptoms were, for example, described in the ancient Indian medical system, Ayurveda, under the name of Kampavata (reviewed in Manyam 1990), and in western culture by Galen 175 AD (Sider and McVaugh 1979). It was not until 1690 that a Hungarian physician Ferenc Pápai Páriz reported the occurrence of all four cardinal symptoms of PD: tremor, bradykinesia, rigor and postural instability (reviewed in Bereczki 2010) in a Hungarian medical text (Páriz 1690). A similar description was then given by James Parkinson over 100 years later in 1817 in his famous ‘Essay on Shaking Palsy’, (Parkinson J. An Essay on the Shaking Palsy. Whittingham and Rowland for Sherwood, Neely, and Jones; London). Approximately 60 years later, the condition was recognized as a medical entity by Jean‐Martin Charcot, who named it after James Parkinson (Charcot 1892; Goetz 1986). Blocq, Marinesco and Brissaud discovered in 1892 and 1893 that lesions in the substantia nigra are associated with PD (Blocq and Marinesco 1892; Brissaud 1893). The seminal work of Constantin Trétiakoff in 1919 supported this view through the analysis of the SN in 54 brains, nine of which were from patients with PD. In all nine brains, Trétiakoff reported the distinct loss of the pigmented neurons in the SN, the presence of neuronal deposits (which he named *corps de Lewy*, referring to their first description in the dorsal nucleus of the vagus nerve by Lewy 1912) and the presence of inflammatory infiltrations (Trétiakoff 1919). All of these characteristics are currently regarded as the major neuropathological hallmarks of PD. Neglecting these findings, neuroanatomists still focused on the striatum as the primary site of the pathophysiology of PD. Then, in 1938, Hassler confirmed the findings of Trétiakoff concomitantly with the observation that the striatum in PD patients is neuroanatomically largely unaltered (Hassler 1938), thus shifting the neuropathological origin of the disease from the striatum to the SN.

### The dopaminergic nature of the neurons in the ventral mesencephalon

From 1938 onward the ‘neuroanatomical history’ of the SN is intimately intermingled with the history of the discovery of the brain's dopaminergic system. Again, these advances were only possible because of the innovation of novel neurochemical techniques that enabled researchers to study chemical compounds, for example, catecholamines, in tissues (Bertler *et al*. 1958). Carlsson showed in his groundbreaking work (in which a conventional experimental approach was used) that the administration of the dopamine precursor L‐3,4‐dihydroxyphenylalanine to mice and rabbits reversed catalepsy. In this study, the catalepsy was induced by treatment with reserpine, resulting in the depletion of catecholamines (Carlsson *et al*. 1957). This reversal was accompanied by the restoration of dopamine levels in the brain (Carlsson *et al*. 1958). Thus, between 1958 and 1960, Carlsson formed the hypothesis that dopamine was a neurotransmitter in the brain and that there might exist a link between catecholamines and movement disorders (Carlsson 1959). This view was substantiated by the findings of Ehringer and Hornykiewitcz (1960), who showed that dopamine is depleted in the striatum of PD patients. Hornykiewitcz's (1963) subsequent finding that dopamine is also depleted in the SN led him to the speculation that the loss of the neurons in the SN is responsible for the loss of dopamine in the brain/striatum of PD patients (reviewed in Hornykiewitcz 1992; Lees *et al*. 2015).

In parallel to these neurochemical technologies implicating dopamine in the pathophysiology of PD, the application of another similarly powerful technology by Carlsson and colleagues revealed the presence of catecholaminergic neuronal groups in the brain (Carlsson *et al*. 1962; Falck *et al*. 1962). In this technology, monoamines are condensed using the formaldehyde vapor, forming an intense fluorophore. Using this technique Dahlström and Fuxe (1964) published their seminal paper detailing the different catecholaminergic cell groups in the medulla oblongata up to the hypothalamus, naming them as cell groups A1–A12. Cell groups A13–A17 are located more rostrally, up to the olfactory bulb (A16) and include the retina (A17). The three adrenalin‐containing groups (C1–C3) in the brainstem were described later following the advent of the newly emerging immunohistochemical techniques. These nowadays indispensable techniques have been used to determine the localization of tyrosine hydroxylase (TH), the rate‐limiting enzyme in the synthesis of catecholamines. In combination with immunohistochemical detection of additional enzymes involved in catecholaminergic synthesis, distinctions could be made between adrenergic, noradrenergic and dopaminergic neurons. Thus, in the mammalian brain, nine dopaminergic cell groups can be distinguished (A8–A16, excluding the dopaminergic neurons in the retina) (Hökfelt *et al*. 1984; Björklund and Dunnett 2007). The nomenclature of the catecholaminergic system that was established in rodents has been retained (in addition to classical neuroanatomical descriptions) and is used across species. Even though the number of catecholaminergic neurons in the different groups varies between species, the basic architecture remains the same. Thus, the A1–A17 nomenclature is specifically useful when analyzing and comparing the system across species and development. Of the nine dopaminergic cell groups, A8–A10 are located in the ventral mesencephalon. As mentioned earlier, these groups are not confined to single anatomical structures but are distributed across structural boundaries and even represent a continuum (reviewed in Bentivoglio and Morelli 2005). However, primary anatomical foci can be named as follows: cells of the A8 group are primarily found in the retrorubral field, and A9 cells are located in the SNc, with some dense aggregation of dopaminergic cells also found in the SNr. A10 cells are located in the ventral tegmental area (VTA). This region is cytoarchitectonically difficult to demarcate and was first described by Tsai (1925); thus, the VTA was originally termed the ‘ventral tegmental area of Tsai’. Extending medially from the VTA, dopaminergic neurons are found in midline structures, such as the central linear nucleus. Whereas the SN (primarily A9 cells) received a great deal of attention because of its selective degeneration in PD (see below), the VTA (primarily A10 cells) drew its share of attention since it was found to play important roles in different neuropsychiatric diseases. This understanding was based on the distinct connectomes of these two regions (see below).

It must be emphasized that the A8–A10 nomenclature relates exclusively to dopaminergic neurons in the ventral mesencephalon, whereas the anatomical nomenclature SN and VTA also includes other neurons with distinct neurotransmitter identities. In addition, the borders are fluid between dopaminergic cell groups. For this reason, they are collectively referred to as the ‘ventral mesencephalic dopaminergic complex’, as suggested by Yetnikoff *et al*. (2014). Quantitatively, the ventral mesencephalic dopaminergic complex encompasses 20.000–30.000 neurons in rodents, 160.000–320.000 in monkeys and 400.000–600.000 neurons in humans. This wide range reflects the increasing complexity of this system in primates (German and Manaye 1993; Nelson *et al*. 1996; Lewis *et al*. 1998; Bentivoglio and Morelli 2005). In rodents, the number of dopaminergic neurons in the SN are equivalent to the number in the VTA (50 : 50). In contrast, in monkeys and humans, the number of dopaminergic neurons in the SN outnumbers those in the VTA (reviewed in Brichta and Greengard 2014). It must be kept in mind that these numbers are only average values for each species. In addition, genetic background is known to affect the number of dopaminergic neurons in the SN. Actually, the number of dopaminergic neurons in the SN differs by up to 33% between inbred mouse strains (Baker *et al*. 1980; Muthane *et al*. 1994). This fact is, unfortunately, often neglected when analyzing animal models of PD.

**Figure 2:**
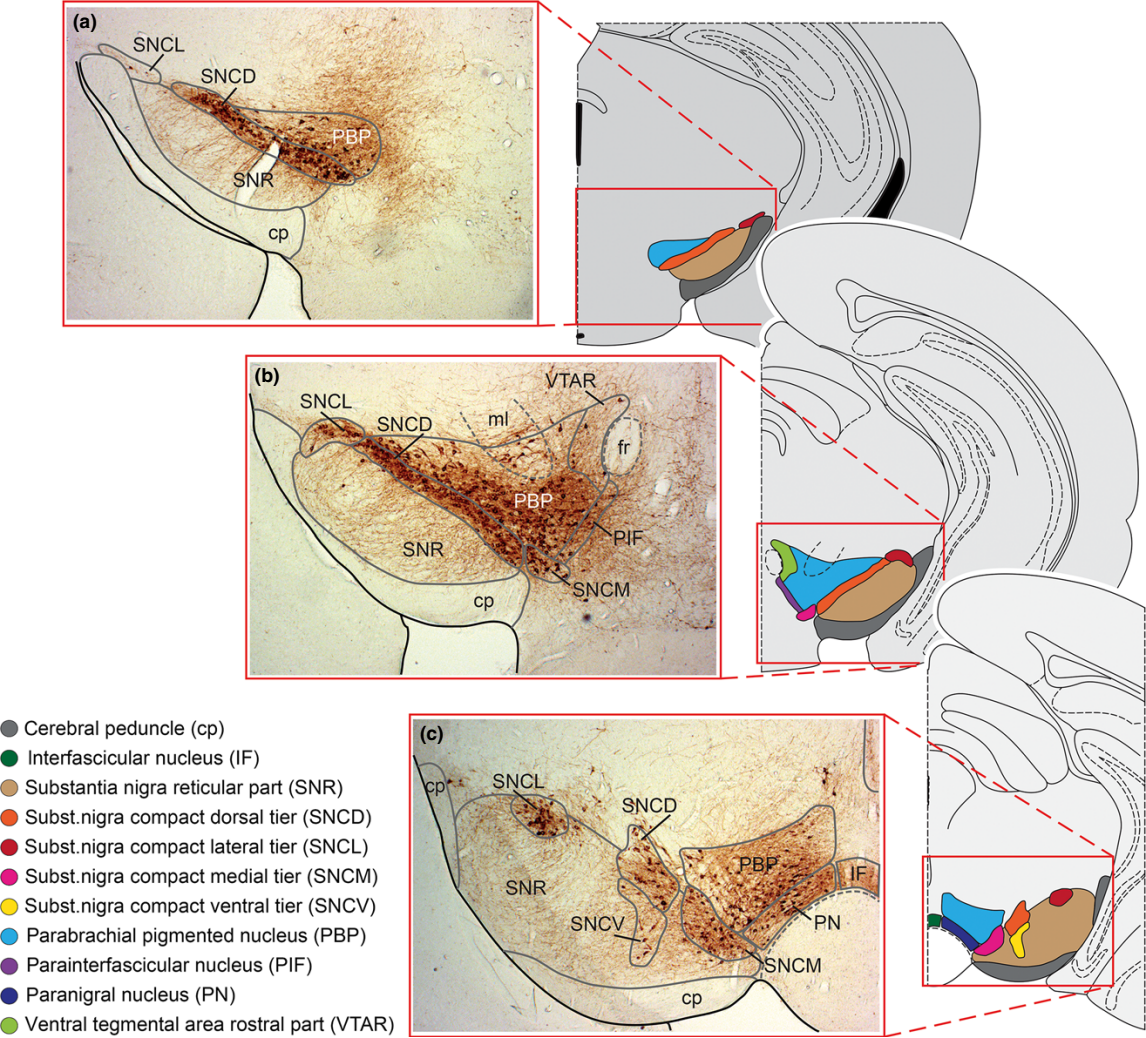
TH‐positive neurons are shown in the murine ventral mesencephalic dopaminergic complex, from rostral to intermediate to caudal (a–c). Neuroanatomically distinct regions are defined according to Fu *et al*. (2012), in which the parabrachial pigmented nucleus (PBP), belonging to the ventral tegmental area (VTA), is clearly demarcated from the substantia nigra pars compacta (SNc), especially the substantia nigra compact dorsal tier (SNCD). Histological sections are mirror images of the colored sketches to indicate the bilateral organization of the complex.

Thus, even though the overall architecture of the ventral mesencephalic complex does not differ across species boundaries, there exists a quantitative difference with respect to dopaminergic neurons located in the different regions. Specifically, there were considerably fewer dopaminergic neurons found in the ventrolateral aspect of the SNc of rats than in primates (Hardman *et al*. 2002). In primates (and rats), these ventrally located dopaminergic neurons are fingerlike extensions of islands into the SNr and, in humans, have been named nigrosomes (Damier *et al*. 1999a,b). This is of special interest in light of the selective degeneration of dopaminergic neurons in PD, since it has been shown (in Hassler 1938) that in humans, neurons which are most affected during the course of the disease are found in the ventrolateral aspect of the SNc, that is, in the nigrosomes (Hassler 1938; Damier *et al*. 1999b; Braak *et al*. 2003; Kordower *et al*. 2013). In contrast, neurons in the VTA and in the dorsal aspect of the SNc are relatively spared by the disease process. Thus, these studies gave early hints of a selective vulnerability among neuronal subpopulations (dopaminergic) in the SNc with respect to the disease process. Interestingly, the ventral tier of dopaminergic neurons succumbing to cell death during PD in humans are now molecularly defined as not expressing the potentially neuroprotective calcium‐binding protein calbindin‐D28K (Damier *et al*. 1999a,b). In mouse, this molecular identity is observed in the dorsal aspect of the SN (SNCD) and a tiny dopaminergic population in the caudal–ventral aspect of the SN (SNCV), as described by Fu *et al*. (2012) (Fig. 2). Thus, the mouse SNCD and the SNCV represent the homologous region to the ventral tier in rats and primates. In contrast, the lateral aspect of the VTA in mouse [specifically parabrachial pigmented nucleus (PBP)] is homologous to the dorsal tier in rats and humans. Thus, in a surprisingly large number of studies in the mouse, this aspect of the VTA (PBP) has been erroneously delineated as the dorsal tier of the SNc. However, for the translation of findings into primates and humans, the correct neuroanatomical assignment of dopaminergic neurons to either the VTA or the SNc (or a subregion thereof) is extremely important when analyzing the number of dopaminergic neurons in genetic and toxin‐induced PD mouse models.

Another issue that must be addressed is the dependency of the extent of dopaminergic neuron loss on disease duration. Earlier studies claimed that the number of neurons lost increases with disease duration (Fearnley and Lees 1991; Greffard *et al*. 2006; Parkkinen *et al*. 2011). However, these studies suffered from either a small amount of patient material and/or the counting of neurons in single sections and not using stereological counting methods. This latter technique was not previously available, and imprecise estimations of neuronal numbers often resulted. The first study using unbiased stereological counting was performed by Ma *et al*. (1997), confirming previous analysis. Still, this study lacked appropriate numbers of brain samples from patients with a long disease duration. Thus, the statistical analyses were confounded by outliers. It was only in 2013 that a study was performed of brains from 17 patients, with the disease duration ranging from 1 to 22 years after the initial diagnosis (Kordower *et al*. 2013). This study again showed that TH‐positive cells are missing in the ventral tier of the SNc; however, in this case, the number of missing neurons was not correlated with disease duration. This neuroanatomical finding – if confirmed – raises the provocative hypothesis that dopaminergic neurons succumb to cell death during the early course of the disease and that ‘clinical deterioration after this time might represent a loss of compensatory mechanisms or degeneration of non‐dopaminergic neurons’ (Nandhagopal *et al*. 2011; Kordower *et al*. 2013). Thus, in this case, the introduction of a new neuroanatomical method (unbiased stereological counting) does not immediately extend our knowledge of the disease but rather raises questions about the previously accepted assertion, namely, the correlation between dopaminergic loss and disease duration. Further studies testing the aforementioned provocative hypothesis are warranted, especially in light of longitudinal clinical imaging data. These analyses use dopamine binding in the striatum as a surrogate marker for loss of dopaminergic neurons in the SNc and show a decline of dopamine binding of 3–5% per year.

Taken together, early research on PD (i.e., from the beginning of the 20th century onward) had revealed a specific, selective vulnerability of neurons in the ventrolateral aspect of the SNc. These reports were later confirmed by studies using modern neuroanatomical techniques, showing that this loss primarily affects dopaminergic neurons. These findings hint strongly toward a diversity among the neurons in the ventral mesencephalic dopaminergic complex. The questions to be solved were: What is the underlying cause of this diversity? Do these neurons project to different target regions? Do they receive distinct inputs and/or are they molecularly distinct?

### Efferents and afferents of the ventral mesencephalon

#### Efferent projections

Even though the substantia nigra *per se* had been identified and its association with PD was proven, its connections within the brain remained a mystery until 1970 (reviewed in Hattori 1993). Early reports from 1895 to 1901, primarily based on lesion studies and subsequent identification of retrogradely degenerating regions, predicted that the cells in the ventral mesencephalon project to the cerebral cortex and the striatum (Monakow 1895; Holmes 1901). Nevertheless, the discussion on the projections of the ventral mesencephalic neurons remained a matter of debate, even when silver methods for the impregnation of degenerating axons and axonal endings had been established. This method, developed by Nauta and Gygax (1954), enabled experimenters to trace fiber connections in the brain. However, it turned out that the method used ‘suppressed, […], the staining of the finer terminal ramifications’ (Nauta 1993). Only a technological improvement of this method developed by Fink and Heimer in (1967) enabled Moore (1970) to unambiguously prove the projection of the ventral mesencephalic neurons to the striatum. This finding was to be expected since about 5 years before – by combining lesion studies and the histofluorescence method – mainly Anden *et al*. (1964) showed that after experimentally destroying the SN, catecholamine fluorescence was missing in the ipsilateral striatum. This result proved that the source of dopamine in the striatum was the dopaminergic neuron population in the ventral mesencephalon that projects through the median forebrain bundle (Moore and Heller 1967).

Thereafter, a further breakthrough in neuroanatomical techniques, the neuroanatomical tracing technology, in combination with immunohistochemical studies led to the beginning of the modern era of neuroanatomy. These technologies allowed a refinement of the projection pattern of the ventral mesencephalon. After the seminal works of LaVail and LaVail (1972) (which described retrograde labeling) and Cowan *et al*. (1972) (anterograde labeling), a multitude of neuronal tracers were applied to reveal the efferent and afferent connections of the dopaminergic neurons in the ventral mesencephalon.

These studies formed the basis for the highly oversimplified view that neurons in the ventral mesencephalic dopaminergic complex project to the dorsal and ventral striatum and the cortex. These projections are referred to as the meso(nigro‐)striatal (to the dorsal striatum), mesolimbic (to the ventral striatum, and e.g., the amygdala) and mesocortical (to different cortical areas) pathways respectively (Bentivoglio and Morelli 2005; Björklund and Dunnett 2007). The mesostriatal pathway was described as being primarily composed by efferents of the A9 group, thus the SN, whereas the latter two were generally described as projecting from the A10 group, thus the VTA. However, Fallon and colleagues elegantly showed in 1978 that distinct dopaminergic target areas in the cortex receive (i) input from dopaminergic neurons of the A9 group in their lateral aspects, (ii) input from the A10 group in their medial aspects and (iii) input from both between their medial and lateral aspects (Fallon and Moore 1978a,b; Fallon *et al*. 1978). Thus, there exists an extensive overlap between the target regions of SN and VTA neurons. The ‘oversimplification’ of the description of these efferent systems was because neurons of the A9 group indeed primarily project to the dorsal striatum (caudate nucleus and putamen, or motor striatum) and to a lesser extent to the ventral striatum (nucleus accumbens and olfactory tubercle, or limbic striatum) and cortical structures. In contrast, the reverse holds true for the A10 group, which is primarily located in the VTA. Thus, for heuristic reasons, it is still valid to distinguish between these three efferent pathways that arise from the ventral mesencephalic dopaminergic complex.

**Figure 3:**
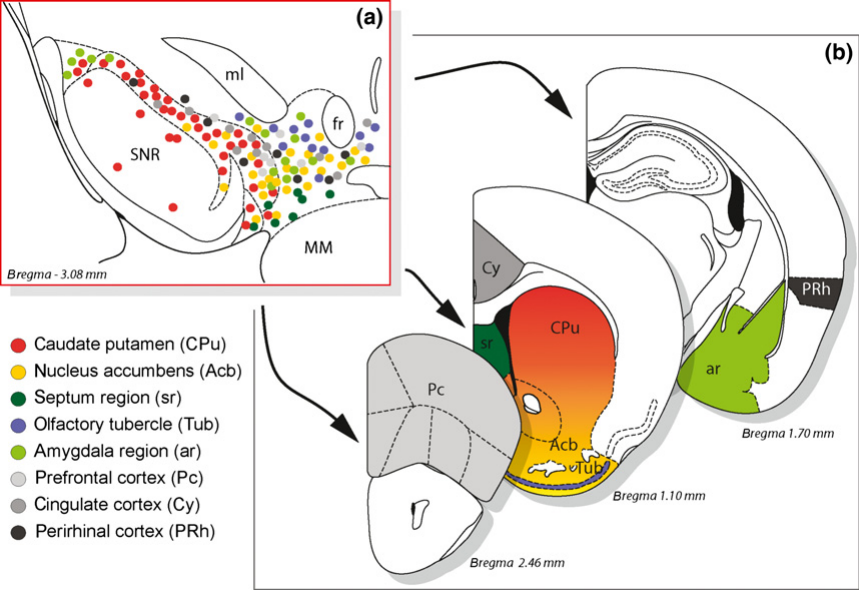
Summary of the projections of neurons in the ventral mesencephalic dopaminergic complex. (a) Neurons in the ventral mesencephalic dopaminergic complex projecting to (b) different telencephalic regions. Neurons and their respective terminal fields are color coded (adapted from Fallon and Loughlin 1995 and Gerfen 2004).

In summary, the major projection areas of A10 and A9 neurons, that is, the VTA and SNc, respectively, can be briefly summarized as follows (reviewed in Haber 2014; Bentivoglio and Morelli 2005; Joel and Weiner 2000) (Fig. 3): (i) dopaminergic neurons of the dorsal medial aspect of the ventral mesencephalic dopaminergic complex, that is, VTA neurons (A10), primarily project to the ventromedial striatum, including the nucleus accumbens (the ‘limbic region’ of the striatum), limbic cortical fields and the amygdala; (ii) neurons in the ventral portion of the complex project to the septum; (iii) dopaminergic neurons in the dorsolateral aspect of the VTA (falsely regarded as the ‘dorsal tier’ of the SNc in mice – see above; Reyes *et al*. 2012; Fu *et al*. 2012) primarily project to the central striatum (the ‘association region’ of the striatum); (iv) neurons projecting to the dorsal striatum (the ‘motor region’ of the striatum, primarily encompassing the putamen) are located predominantly in the SNc (the ventral tier of the SNc in primates, and the SNc in mice); (v) neurons in the substantia nigra pars lateralis project to the amygdala. Thus, regarding the projections to the striatum a specific medial to lateral, anterior to posterior and inverted dorsal to ventral topography exists (Pan *et al*. 2010; Fallon and Moore 1978b; reviewed in Joel and Weiner 2000; Haber 2014). However, although a topography is present, due to the fact that individual dopaminergic neurons have widespread striatal projections, a considerable overlap in projection targets exist (Parent and Parent 2006).

In addition to this regional spatial organization, the nigrostriatal projection also largely respects the compartmentalization of the striatum, defined by the patch/striosome and matrix compartments. These two compartments are defined by their differential neurochemical composition (e.g., higher expression in patch/striosomes of substance P) and their differential cortical input and striatofugal output organization (Crittenden and Graybiel 2011). Until approximately 10 years ago, it was thought that the projections of the dorsolateral aspect of the VTA terminate solely in the matrix of the striatum and that the projections from the SNc terminate solely in the patch/striosome compartment (for review, see Bentivoglio and Morelli 2005). Thereafter, using juxtacellular labeling and single‐cell tracing, this oversimplified view had to be revised in the sense that single dopaminergic neurons of the ventral mesencephalon innervated both striosome and matrix compartments. However, each neuron's arborization still tends to favor one of these compartments (Matsuda *et al*. 2009). The functional significance of these compartments was long unknown, but they were thought to serve different aspects of motor activity and behavior. This view was substantiated by an elegant study implicating a prefronto‐striosomal circuit specifically in cost–benefit decisions (Friedman *et al*. 2015) in rats. In addition, cost–benefit decisions in primates have been suggested to be governed by a similar prefronto‐striosomal circuit (Amemori and Graybiel 2012). As this report was limited to the analysis of the cortical input to the dorsomedial striatum, it might not fully describe striosomal functions. Specifically, it would be interesting to relate these function(s) to the afferents from the ventral mesencephalic dopaminergic complex, as has been reported recently by Brimblecombe and Cragg (2015). These authors showed that neuropeptide substance P – expressed at high levels in the striosomes – modulates DA transmission in this compartment. Thus, although single dopaminergic neurons do not respect all neuroanatomically defined boundaries within the striatum, DA transmission is governed by these boundaries. This fact may have significant implications for the function of the respective neuronal circuits and, thus, behavioral and/or pathological outcomes.

Taken together, these previous results indicate that anatomically distinct subsets of dopaminergic neurons within the A9–A10 groups (i.e., the SN and the VTA respectively) project to specific brain regions, adding a further level of diversity with respect to the functionality to dopaminergic neurons. The projections are topographically organized and the topography is conserved across species. Thus, within the ventral mesencephalic dopaminergic complex, neurons are distinguished based on their projection pattern(s). This raises the question of whether distinct sets of dopaminergic neurons are defined not only by their anatomical location and efferent projections, but also by their afferent projections, that is, their innervation.

#### Afferent connections

With respect to the innervation of neurons in the ventral mesencephalic dopaminergic complex, specifically the VTA, widespread input from cortical regions, the striatum, the globus pallidus, thalamus, subthalamic nucleus, superior colliculus, raphe nuclei and some brainstem nuclei is firmly established (Geisler and Zahm 2005; as reviewed in Oades and Halliday 1987; Yetnikoff *et al*. 2014). Of special interest in this respect is the reciprocal connection between the ventral mesencephalic dopaminergic complex and the striatum. The ventral striatum projects to the VTA, and dopaminergic neurons in the VTA project to the ventral striatum. However, because of their widespread terminal fields (see above), these latter projections also innervate the associative striatum. In turn, associative striatum neurons project back to the more dorsal aspect of the SNc and in part to the ventral aspect of the SNc. Neurons in the ventral aspect of the SNc project back to the dorsal striatum. In this way, a lateral spiraling of striato‐nigral‐striatal connectivity is established, which is hypothesized to be the underlying substrate for the development of drug addiction, progressing from reward states to habitual states, that is, motor habits (Haber *et al*. 2000).

It also must be mentioned that, in terms of the progress that has been made in understanding the dopaminergic system via the introduction of new technologies, novel approaches can lead to challenges of previously existing views of the system. For example, a combination of anatomical techniques with electrophysiological recordings and optogenetics (Chuhma *et al*. 2011) initially raised doubts as to the existence of projections from striatal neurons to dopaminergic neurons in the SNc (Somogyi *et al*. 1981; Bolam and Smith 1990). However, this controversy was soon laid to rest following the introduction of another new technology, namely, monosynaptic circuit tracing with glycoprotein‐deleted rabies viruses (for review, see Callaway and Luo 2015). The technique was developed by Wickersham *et al*. (2007a,b), and since this time, has been applied to overcome the limitations of conventional neuroanatomical tracing techniques. One major limitation was the impossibility (or highly labor‐intensive way via electronmicroscopical studies) of unambiguously assigning synaptic inputs to specific neurons, that is, distinguishing between inputs to dopaminergic and non‐dopaminergic neurons in the SNc. The technique developed by Wickersham *et al*. (2007a,b) combines the Cre/loxP gene expression system with rabies‐virus‐based trans‐synaptic retrograde tracing. This method can be extended using specific combinations of genetic and tracing approaches to identify dopaminergic neurons that project to a specific site. This technique also allows the concomitant identification of the inputs to these neurons from the rest of the brain, which is known as the ‘cell‐type‐specific tracing of the relationship between input and output’, or cTRIO method (Schwarz *et al*. 2015). Furthermore, in combination with optical clearing and light‐sheet microscopy, it is possible to generate whole‐brain maps of direct inputs to the neurons of the ventral mesencephalic dopaminergic complex (Watabe‐Uchida *et al*. 2012; Beier Kevin *et al*. 2015; Lerner *et al*. 2015; Menegas *et al*. 2015). These maps in general verified the different inputs to the ventral mesencephalic dopaminergic complex that were established using simple neuroanatomical tracing techniques. In addition, these novel methods proved that these inputs indeed terminate on dopaminergic neurons. The inputs specifically to the dopaminergic neurons of the SNc (those succumbing to cell death during PD) were recently studied by Lerner *et al*. (2015). These authors specifically looked into the reciprocal connection of the SNc with the striatum and verified the existence of a topographically organized connection. In addition, using optogenetic techniques, it was shown that the input from more lateral portions of the dorsal striatum is much stronger than from medial portions. This distinction might be important in the context of PD. Specifically, the more lateral aspect of the striatum is affected in PD as a result of its innervation by the ventral‐lateral aspect of the SNc, in which dopaminergic neurons are lost. Thus, multi‐technological approaches in animal models (reviewed in Lerner *et al*. 2016) are now essential given how they improve our understanding of the ongoing disease process in specific dopaminergic neuronal circuits. Such protocols are especially important with respect to the selective degeneration of the affected circuit; this damage initiates in the ventral SNc and thereby increases the relative activity of the medial dopaminergic cell groups (discussed in Reyes *et al*. 2013a). Understanding these ‘diseased’ circuitries might also lead to insights into the pathophysiology of co‐morbid diseases, such as apathy (Pagonabarraga *et al*. 2015), and unwanted side effects of current L‐3,4‐dihydroxyphenylalanine therapies, such as impulsive–compulsive behaviors (Pirritano *et al*. 2014). Although this review primarily addresses the connectome between the dopaminergic neurons of the ventral mesencephalon (with a focus on the SNc) and the striatum, these neurons have widespread connections (afferents and efferents) throughout the brain, including feed‐forward and feedback connections to other components in the basal ganglia. These pathways add further complexity to the composition and function of the ventral mesencephalic complex.

Taken together, the neuroanatomical studies presented strongly suggest the existence of subtypes of dopaminergic neurons in ventral mesencephalic regions, not only in terms of their localization, but also their connectomes. These differences are attributable in part to heterogeneity among the neurons of the VTA (A10 group) and the SN (A9 group). However, it is increasingly evident that heterogeneity also exists within these anatomical structures, which is reflected in the finding of dopaminergic populations with distinct neurotransmitter and molecular identity.

## Diversity of neurons in the ventral mesencephalic dopaminergic complex

### Diverse neurotransmitter content

Awareness of the diversity of dopaminergic neurons in the ventral mesencephalic dopaminergic complex has increased in recent years. In addition to insights into neuroanatomical diversity (e.g., their morphology, localization and both efferent and afferent connections), electrophysiological and optogenetic studies have substantiated the existence of diverse dopaminergic neurons in this complex. These studies clearly showed that distinct dopaminergic populations mediate distinct behaviors. In addition, these previous studies confirmed that subpopulations of dopaminergic neurons might also be distinguished based on distinct co‐release of neurotransmitters, such as glutamate and/or GABA. This form of signaling is referred to as multiplexed neurotransmission (Tritsch *et al*. 2012; Zhang *et al*. 2015b; Root *et al*. 2014; El Mestikawy *et al*. 2011; reviewed in Trudeau *et al*. 2014; Barker *et al*. 2016; Tritsch *et al*. 2016). Concerning the capability of co‐release of glutamate by dopaminergic neurons, it must be noted that between 5% and 20% of dopaminergic neurons in the VTA express vesicular glutamate transporter‐2. In the SNc, only a small fraction in the lateral portion exhibits this double labeling (Yamaguchi *et al*. 2007, 2011, 2015). These findings are in close agreement with recent optogenetic studies showing that glutamate‐mediated synaptic currents can be easily detected in the ventral striatum but only rarely in the dorsal striatum (Stuber *et al*. 2010; Tecuapetla *et al*. 2010). In contrast, GABA‐mediated synaptic currents can be readily detected in the dorsal striatum, indicative of GABA activity in dopaminergic neurons of the SNc. Interestingly, with the exception of a small fraction of dopaminergic neurons, these neurons do not express GABA synthetic enzymes nor the vesicular GABA transporter (vGAT), implicating non‐canonical GABA synthesis and packaging (Tritsch *et al*. 2012, 2016). Concerning the latter possibility, a recent study revealed that VMAT2 is necessary for the GABAergic transmission from dopaminergic neurons, thus raising the possibility that dopamine and GABA are not only co‐released, but also co‐packaged into dopaminergic vesicles in SNc neurons (Tritsch *et al*. 2012). This finding has been substantiated by Kim *et al*. (2015), who also demonstrated a novel GABA synthesis pathway mediated by aldehyde dehydrogenase 1a1 (ALDH1a1). This enzyme is highly expressed in the dopaminergic neurons in the SNc (see below) and is necessary for the co‐release of dopamine and GABA.

The high degree of complexity of this multiplexed neurotransmission of dopaminergic neurons is also reflected by the fact that even though glutamate can be released from a dopaminergic axon, it is normally not released at the same site or from the same synaptic vesicles as dopamine (Zhang *et al*. 2015b). This ‘differential spatial release’ might be one explanation why the modulatory effect of glutamate and/or GABA co‐release differs dramatically between the ventral and dorsal striatum and target cells (i.e., inhibitory spiny stellate cells and cholinergic interneurons) as well as between forebrain targets of dopaminergic neurons (Chuhma *et al*. 2011, 2014; Mingote *et al*. 2015). Thus, research into multiplexed neurotransmission adds another layer of complexity onto the diversity of dopaminergic neurons, extending the diversity to the synaptic connections by which dopaminergic neurons elicit diametrically opposite responses from different target neurons. The functional significance of this multiplexed neurotransmission is an intense focus of research given that it is very likely involved in the transmission of precise temporal signals and dramatically enhances the dynamic range of the dopaminergic signals necessary to regulate diverse behavior, for example, addiction. Toward this end, Kim *et al*. (2015) showed that ALDH1a1, the enzyme responsible for the production of GABA in dopaminergic neurons, also modulates alcohol intake and preference behavior. Thus, the functional significance of the co‐release of GABA and DA from dopaminergic neurons may be found, at least in part, in the fine‐tuning and regulation of the development of addictive behavior.

Notably, with respect to the functional diversity of dopaminergic neurons, researchers have primarily focused on the dopaminergic neurons in the VTA (e.g., Lammel *et al*. 2008, 2011, 2012; Stamatakis *et al*. 2013). Only recently has the functional diversity between dopaminergic neurons of the SNc been appreciated (Matsumoto and Hikosaka 2009; Lerner *et al*. 2015; reviewed in Roeper 2013). However, this dopaminergic population urgently requires further study, given that the diverse functions and/or molecular identity of these neurons might explain their selective vulnerability in PD.

### Diverse gene expression

**Figure 4:**
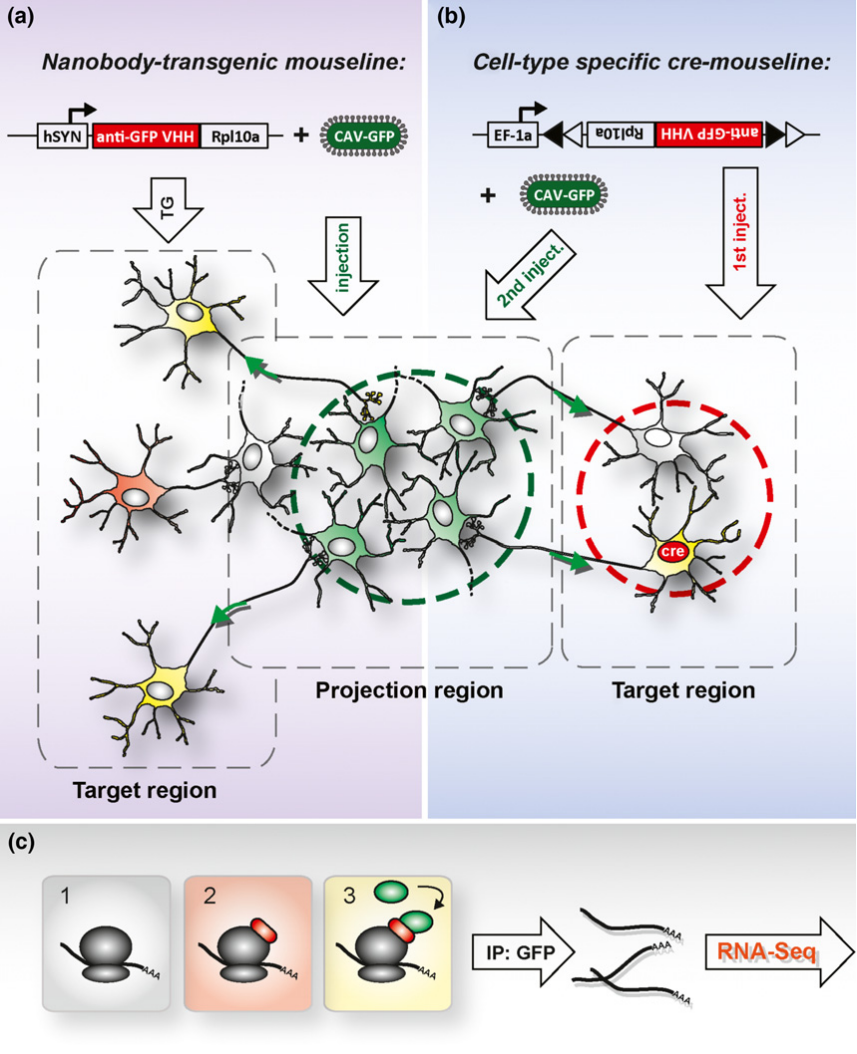
The functional principle of retro‐TRAP (translating ribosome affinity purification utilizing viral retrograde tracing). (a) Transgenic mice are generated that over‐express an anti‐GFP nanobody fused (anti‐GFP VHH) to the ribosomal subunit Rpl10a. This construct is under the control of the pan‐neuronal human synapsin promoter (hSYN), and the mice are injected stereotactically with a retrograde tracing virus (canine adenovirus 2) expressing GFP (CAV‐GFP) into the desired projection region. Neurons projecting to virus‐transduced target cells (green) receive the GFP trans‐synaptically (yellow), while those projecting to non‐transduced cells (gray) remain GFP negative (red). (b) As these cells (red) lack the expression of GFP (2), affinity purification of GFP will only result in purification of RNA from double‐positive neurons (yellow, 3), which comprise the GFP‐nanobody–ribosome‐RNA complex. (c) Advances in specificity allows for the combination of retro‐TRAP with cell‐type‐specific Cre‐driver mouse lines. For this, an adeno‐associated virus bearing a Cre‐dependent anti‐GFP nanobody fusion protein (anti‐GFP VHH) must be injected into the desired target region of a Cre‐driver mouse line. The expression of the nanobody fusion protein is restricted to Cre‐positive neurons in the injection area. After the subsequent injection of the retrograde tracing virus (CAV‐GFP) into the projection area, RNA can be affinity purified exclusively from neurons with the desired molecular characteristics (Cre‐dependent), from a certain region (AAV injection dependent), or with certain connectivity properties (CAV injection dependent) (adapted from Ekstrand *et al*. 2014).

Electrophysiological and optogenetic studies have revealed the diversity of dopaminergic neurons at the functional level. These results were paralleled by an increasing number of reports on differential gene and protein expression being used to distinguish between the VTA and SNc as well as neuronal populations within these regions. These reports therefore describe neuronal diversity at the molecular level and provide the first insights into the molecular characteristics that could underlie the selective vulnerability of dopaminergic neurons in the SNc in PD (reviewed in Anderegg *et al*. 2015; Brichta and Greengard 2014; Veenvliet and Smidt 2014; Double *et al*. 2010; Korotkova *et al*. 2004). Thus, there is now an extensive body of literature describing the differential gene expression between different dopaminergic populations. However, it must be noted that despite the functional differences of the dopaminergic neurons in the ventral mesencephalon, the gene expression signatures of these populations are extremely similar, that is, 97–99% of the genes expressed in the ventral mesencephalic complex are expressed in both the VTA and the SN (Grimm *et al*. 2004; Chung *et al*. 2005; Greene *et al*. 2005). However, the remaining 1–3% might play essential roles in determining the differential vulnerability of dopaminergic neurons in the SNc. These studies, together with a wealth of immunohistochemical data, revealed that the differentially expressed genes (generally between the VTA and SNc) could roughly be grouped as follows: genes implicated in neuronal dopamine biology [e.g., Slc6a3 (DAT), DRD1, DRD2 and ALDH1a1], neuronal activity [e.g., KCNJ6 (GIRK2), KCNN3, calcium‐binding proteins], neuronal survival (e.g., brain‐derived neurotrophic factor; glial cell line derived neurotrophic factor) and transcription factors (specifically those implicated in the specification and differentiation of dopaminergic neurons (e.g., PITX3, OTX2, SOX6). Specifically, the latter set might underlie the distinct molecular phenotypes of dopaminergic neurons. Two notes of caution must be made. First, the list of known differentially expressed genes is highly biased. Specifically, it is generally genes that are implicated in dopaminergic neuronal biology/function that have been analyzed in terms of their expression patterns. Second, although regional expression differences have been reported for certain genes, these differences do not exhibit an ‘all or nothing’ pattern; rather, there are differences in expression levels that have been described in the literature as either ‘strong’ versus ‘weak’ expression or as ‘enrichment of gene expression’. Therefore, it is highly important to determine whether the terminology of, for example, ‘enrichment’ is indeed because of a general higher expression in all dopaminergic neurons of the given region or is because of only a subset of neurons in this region expressing the specific gene. This latter situation was observed for the expression of engrailed genes in both the VTA and SN (Simon *et al*. 2001). Thus, the technical progress, made primarily in the last year, in terms of applying single‐cell transcriptomics to neurons (Tang *et al*. 2009; Macosko *et al*. 2015; Usoskin *et al*. 2015; Zeisel *et al*. 2015) will be crucial for revealing the transcriptomes of single dopaminergic neurons. These data will allow the identification of molecularly defined subtypes. The next step will be to evaluate and explore the relationship between the molecular signature of a specific subtype with its location, morphology, connectivity and excitability. Indeed, such an approach has proved to be successful in the cortex, correlating excitability with molecular signatures of neuronal subtypes (Fuzik *et al*. 2015). In addition, the advent of retro‐TRAP technology now allows for correlations between specific molecular subtypes and its connection patterns (Ekstrand *et al*. 2014; Nectow *et al*. 2015) (Fig. 4).

Concerning the dopaminergic system, a first step was made toward single‐cell transcriptome analysis in a study of the expression of just 96 selected genes (Poulin *et al*. 2014). These genes are known to be involved in dopaminergic biology/function throughout the ventral mesencephalic dopaminergic complex. This analysis revealed (by hierarchical clustering) two primary molecularly defined clusters of dopaminergic neurons (DA^1^ and DA^2^). These clusters were again subdivided into two (DA^1A/1B^) and four subtypes of dopaminergic neurons (DA^2A‐D^) (Poulin *et al*. 2014; Anderegg *et al*. 2015). Interestingly, the two main clusters separated the dopaminergic neurons within the SN from those in the VTA. In addition, subsequent subdivision of these primary clusters into six subtypes revealed that these populations can be assigned to distinct neuroanatomical regions in the ventral mesencephalic dopaminergic complex. Thus, the two subtypes (DA^1A^ and DA^1B^), which are both characterized by their expression of the Sox6 transcription factor, are restricted to the SNc and the dorsolateral aspect of the VTA, that is, the PBP (as defined by Fu *et al*. 2012). This result is consistent with the requirement of this transcription factor in the development of these dopaminergic populations (Panman *et al*. 2014). The DA^1A^ subtype is predominantly found in the SNc, which represents the most vulnerable dopaminergic neurons in the context of PD. Interestingly, this subtype expresses the highest levels of the ion channel Kcnj6 (Girk2), which has been proposed to confer a specific vulnerability to SNc dopaminergic neurons in toxin models of PD (Liss *et al*. 2005). In addition, the unique expression of Otx2 in DA^2^ (primarily DA^2B^ and DA^2C^) (Anderegg *et al*. 2015) again is consistent with previous studies (Di Salvio *et al*. 2010b). Specifically, this transcription factor is proposed to antagonize the vulnerability of neurons to MPTP (Di Salvio *et al*. 2010a). However, this single‐cell study also described some findings that were contradictory to previous reports. For example, the subtype DA^1A^, which is primarily found in the SNc and is lost upon MPTP treatment (Poulin *et al*. 2014), is also characterized by the expression of ALDH1a1. At first glance, this result is in sharp contrast to earlier studies, in which ALDH1a1 was assigned a neuroprotective function based on its oxidation of a potential neurotoxic metabolite of dopamine (DOPAL), which promotes the formation of α‐synuclein oligomers (reviewed in Cai *et al*. 2014a). Thus, cell death induced by α‐synuclein oligomerization is prevented by ALDH1a1. In contrast, cell death induced by other neurotoxins such as MPP+ and glutamate was unaffected by the loss of ALDH1a1 (Liu *et al*. 2014). Thus, ALDH1a1 might preferentially protect against the α‐synuclein‐mediated loss of mesencephalic dopaminergic neurons, which represents one of the proposed mechanisms of neuronal cell death in PD. In addition, the presence of such a neuroprotective mediator in highly susceptible dopaminergic populations might be insufficient to prevent the effects of additional pathogenic events during disease progression. Furthermore, the primary role of ALDH1a1 in this dopaminergic neuronal population might not be neuroprotection, but rather the generation of GABA via a non‐canonical synthesis pathway, as described earlier (Kim *et al*. 2015).

In addition to confirming the existing data on differential gene expression across the ventral mesencephalic dopaminergic complex, this single‐cell study and those that follow will form the basis for hypotheses concerning the specific vulnerability of SNc neurons. An example in this respect is the predominant expression of Fzd1 in the primary cluster DA^2A‐D^ (primarily neurons in the VTA) (Poulin *et al*. 2014). Fzd1 is a Wnt1 receptor that has been shown to be instrumental for the development of dopaminergic neurons (Prakash *et al*. 2006, reviewed in Wurst and Prakash 2014). Interestingly, the pharmacological or genetic ectopic activation of the Wnt‐signaling pathway in the adult mesencephalon has been shown to protect dopaminergic neurons from both cytotoxic insults (L'Episcopo *et al*. 2011a,b) and from degeneration upon loss of the transcription factor En‐1. The latter effect is most likely a result of the reactivation of a gene cascade that is active during development (Zhang *et al*. 2015a). A neuroprotective role of the Wnt1 pathway is at first glance counterintuitive given that this ligand is not expressed in the adult mesencephalon. However, Wnt1 becomes expressed in reactive astrocytes upon injury of this region (reviewed in L'Episcopo *et al*. 2014). Thus, it is tempting to speculate that dopaminergic neurons within DA^1^ (specifically subtype DA^1A^) are more vulnerable to toxic insults (and perhaps to the PD disease process) given that they do not express Fzd1. Interestingly, En1, Wnt1, Otx2, Aldh1a1 and Sox6 are all implicated in the development of the respective subtypes of dopaminergic neurons (Bodea and Blaess 2015; Veenvliet and Smidt 2014). Thus, it is possible that one role of these genes in dopaminergic neurons is to protect the respective subpopulation upon injury by reactivating developmental programs (Zhang *et al*. 2015a).

Another interesting facet of PD biology that should be addressed is the expression of genes known to be associated with genetic forms of PD. Over the last decade, several genes have been implicated and associated with the pathoetiology of the disease (reviewed in Verstraeten *et al*. 2015; van Brug *et al*. 2015). Expression studies have shown that these genes are rather ubiquitously expressed throughout the brain, most even in glial populations (Petersen *et al*. 1999; Stichel *et al*. 2000; Bader *et al*. 2005; Blackinton *et al*. 2007; Pham *et al*. 2010; Giesert *et al*. 2013). The study by Poulin *et al*. (2014) confirmed that neither the expression of α‐synuclein, Parkin, DJ‐1, Pink1, Atp13a2 nor Lrrk2 in neurons of the ventral mesencephalon is restricted to a specific dopaminergic subpopulation. One of the only significant findings in this regard was a high expression of α‐synuclein in subtypes DA^1A^ and DA^2C^. Thus, it is unlikely that the expression of mutated forms of PD‐associated genes underlies the selective vulnerability of dopaminergic neurons in the SNc. This hypothesis is also supported by the promiscuous involvement of these genes in numerous essential cellular mechanisms. However, their dysfunction might enhance a pre‐existing bias in sensitivity to neurodegeneration, a proposal that is in agreement with the multiple hit hypothesis of PD (Sulzer 2007).

Taken together, the technology that is now available to identify molecular signatures at the single‐cell level, in combination with electrophysiology, tracing technologies and optogenetics, will allow for the full characterization of dopaminergic neuron subtypes in the ventral mesencephalic complex. This careful characterization must be followed by functional studies to reveal the relevance of single genes or gene networks in terms of dopaminergic cell‐type‐specific functions and vulnerability in PD (i.e., whether these genes confer susceptibility or neuroprotection to the respective dopaminergic neuron).

### Further diversification

Adding to the complexity of molecularly diverse dopaminergic neuronal population in the ventral mesencephalic complex is the increasing evidence that, in addition to gene expression, other cellular and biochemical characteristics are differentially regulated within the complex. For example, post‐transcriptional modifications that regulate the activity of specific gene products might differ between different populations of DA neurons. One prominent example is the glycosylation of Slc6a3 (indicative of the active form of the transporter), the expression of which has been shown to be higher in the SNc than in the VTA (Afonso‐Oramas *et al*. 2009; Di Salvio *et al*. 2010a). Another possibility is diversity concerning bioenergetic parameters. Indeed, it has recently been shown that SNc neurons are near their maximal capacity at basal state respiration, exhibiting a close to three‐fold higher cellular respiration rate compared to VTA neurons. This elevated respiration was shown to be because of SNc dopaminergic neurons having more complex axons and a higher density of axonal mitochondria than VTA neurons. Thus, coming back to neuroanatomical features, it can be speculated that this specific morphology might underlie the vulnerability of SNc neurons in that their increased basal energy demands might generate higher oxidative stress (Pacelli *et al*. 2015). It would also be interesting to know whether the differentially expressed gene deleted in colorectal cancer (DCC), an axon guidance molecule expressed at high levels in SNc dopaminergic neurons (Osborne *et al*. 2005; Reyes *et al*. 2013b), is involved in the generation and maintenance of the elaborate axonal arborization of these neurons. It is known that DCC regulates the guidance of SNc and VTA dopaminergic axons to the striatum via its interaction with netrin (Li *et al*. 2014). However, if there is a bias of DCC expression during development (or of other guidance molecules implicated in dopaminergic neuron axon pathfinding; Prestoz *et al*. 2012) between neurons that project to the dorsal and ventral striatum remains to be determined.

In this context, the question arises of whether the diversity of dopaminergic neurons is genetically programmed. An extensive body of work has been dedicated to unravel the molecular mechanism involved in the specification, differentiation and maintenance of dopaminergic neurons in the ventral mesencephalon. These studies have led to the identification of signaling and transcriptional networks that control the specification of a general dopaminergic fate during development (recently reviewed in Arenas *et al*. 2015; Wurst and Prakash 2014). In contrast, how the diverse dopaminergic subtypes in the ventral mesencephalic complex are established during brain development is only starting to be unraveled. The state‐of‐the art in this research field was recently excellently reviewed by Veenvliet and Smidt (2014) as well as Bodea and Blaess (2015). These authors stated that both genetic and experience‐dependent processes are involved. Specifically, the latter supports the notion that epigenetic modifications, expression of non‐coding RNA and miRNAs, all of which having been implicated in the pathogenesis of PD (reviewed in Feng *et al*. 2015), might also display regional variations within the ventral mesencephalic dopaminergic complex. Thus, diversity of dopaminergic neurons and the subtype specific vulnerability of dopaminergic neurons in PD might be determined by these factors as well.

Taken together, the technological advancements in recent last years have allowed the field to begin to characterize single neurons in their specific brain microenvironments. These efforts will certainly increase our understanding of the function of neurons in the ventral mesencephalic dopaminergic complex in health and disease. Still, it remains an open question of whether single‐cell studies will reveal even more diversity in the dopaminergic neurons than is currently known. We may be forced to acknowledge that every neuron represents a singular, unique entity. This hypothesis is supported by recent omics studies in the brain. It has been shown that transcriptomes of neurons in particular are highly dynamic and exhibit a high number of genes with bimodal on or off expression (Lovatt *et al*. 2014; Dueck *et al*. 2015). The dynamism of neuronal transcriptomes is thought to depend in part on the developmental stage, neuronal activity, connectivity and tissue environment. In addition, it is by now established that single neurons might have different genomes. Genomes of individual cells can vary in terms of duplications, deletions and single nucleotide variants, which are highly likely to affect the transcriptome (Kaushal *et al*. 2003; McConnell *et al*. 2013; Cai *et al*. 2014b; Lodato *et al*. 2015). These differences are thought to accumulate over time and might be introduced by mobile elements (transposons) within the genome, which can change their own position (reviewed in Erwin *et al*. 2014). In addition, the transcriptome can be changed by the epigenome, which again has been proven to be highly dynamic in neurons (Guo *et al*. 2014). Thus, it may ultimately be determined that each dopaminergic neuron is molecularly unique. This dynamic molecular variability, however, might form the underlying mechanisms for higher level functioning of neural circuits (Dueck *et al*. 2016).

## The need for new tools

As described earlier, recent advances in our understanding of the functionality of the dopaminergic neurons in the ventral mesencephalon have been made by applying new technologies, such as monosynaptic tracings, optogenetics and single‐cell transcriptomics. However, these technologies all heavily rely on the availability of genetically modified mice to address and/or manipulate the different subtypes of dopaminergic neurons in this region. In this context, conditional mutagenesis such as the Cre‐LoxP system has been the primary tool (Rajewsky *et al*. 1996). This system is based on the interaction of a Cre recombinase (expressed in Cre‐driver mice) with so‐called loxP sites, which are recognized by the Cre recombinase and can be introduced into any DNA sequence. The key factor determining the specificity of this system is the choice of the promotor under which the Cre recombinase (Cre) is expressed. When Cre is expressed under a specific neuronal subtype promoter, the genetic manipulation (i.e., the conditional knockout of a gene and/or the activation/inhibition of a reporter) only takes place in this specific neuronal cell population (this process is termed conditional mutagenesis). In addition to introducing this spatial dimension into the genetic manipulation, a temporal dimension can be introduced using a tamoxifen‐inducible Cre (CreERT2). Thus, Cre can be activated in a temporally specific manner at any time during development into adulthood. In this way, the cell‐type‐specific function of genes can be elucidated and the functional manipulation of molecularly defined cell types is possible. However, the results of these studies are difficult to interpret when using promoters of genes that are expressed in different subtypes of dopaminergic neurons, such as Sox6 (see above). In addition, as the genetic manipulation induced by Cre is a discrete (i.e., all‐or‐nothing) event, low expression of Cre can still introduce the desired genetic manipulation, rendering a distinction between cells with high and low marker gene expression impossible. Another confounding factor is that many Cre‐driver lines are based on the expression of Cre under the control of promoters within a transgene that has been randomly integrated into the genome. These Cre‐driver lines often suffer from unspecific expression of the recombinase in multiple off‐target cell types. This situation can confound the results of the highly sophisticated new technologies that are used to determine cell‐type‐specific function. Therefore, it will be necessary to (i) restrict the usage of Cre‐lox systems to Cre‐driver lines that have been extensively characterized with respect to their cellular expression patterns; (ii) extend the availability of existing Cre‐driver lines in defined genetic backgrounds (to control for genetic background effects); (iii) extend Cre technology toward intersectional Cre expression if specific neuronal populations can only be distinguished by distinct combination(s) of gene expression patterns (i.e., drive Cre expression using two different promotors with overlapping expression); and/or (iv) combine Cre with Flp‐ or Dre‐recombinase technology (reviewed in Pupe and Wallen‐Mackenzie 2015; Branda and Dymecki 2004). Thus, new and highly characterized recombinase driver lines are necessary to resolve the specific function of neuronal subtypes in the ventral mesencephalon. Indeed, generating new Cre‐driver lines to expand tissue‐ and cell‐type‐specific conditional mutagenesis is one of the major tasks of the EUCOMMTools Consortium in the framework of the International Knockout Mouse Consortium (IKMC: http://www.mousephenotype.org) (Rosen *et al*. 2015). This consortium provides the scientific community with approximately 200 Cre‐driver lines that express Cre under cell‐type‐specific promoters. The consortium also offers powerful genetic tools, such as cell visualization and gene manipulation cassettes. The latter are tailored to be introduced in IKMC alleles (also in combination with clustered regularly interspaced short palindromic repeats (CrispR) technology), allowing fast and reliable generation of transgenic mouse lines. Thus, this genetic IKMC toolbox is an ideal resource for the types of multifaceted functional studies necessary to unravel the functional diversity of various neuronal populations (e.g., subtypes of dopaminergic neurons in the ventral mesencephalon) and/or for generating new animal models for PD.

Taken together, these new tools will further increase our understanding of the function of the ventral mesencephalic dopaminergic neurons in the context of both health and disease. However, it must be noted that although mouse models are highly valuable and important in delineating molecular mechanisms underlying PD pathology, they cannot recapitulate every aspect of PD observed in primates and humans (for review, see Pickrell *et al*. 2013; Smith *et al*. 2012; Dehay *et al*. 2016; Hatami and Chesselet 2015; Gubellini and Kachidian 2015; Dawson *et al*. 2010). This limitation might be because of species differences, specifically with regard to the vulnerability of dopaminergic neurons in PD. There are numerous potential reasons for these differences, including the short life span of mice, species‐specific molecular characteristics and differences in the neuronal circuits in dopaminergic neurons in the ventral mesencephalon. Thus, being able to dissect cell‐type‐specific function in primates is of utmost importance with respect to understanding PD pathology. It might soon be possible to realize this goal with the advent of genome editing using CrispR/Cas9 technology. Using this technique, *in vivo* genome editing is no longer limited to mice (Niu *et al*. 2014; Chen *et al*. 2015; Tu *et al*. 2015). Therefore, it is possible that, similar to genetic mouse models, primate models can be generated to express recombinases or optogenetic tools for dissecting cell‐type‐specific functions. Although it is very tempting to use such technologies in primates to advance our understanding of the functionality and diversity of dopaminergic neurons in the ventral mesencephalon, the highest ethical standards must be applied. Each genetic manipulation in primates must be carefully judged with respect to whether the benefit of the thereby newly acquired knowledge still outweighs the ethical concerns.

## Conclusion

In summary, considering the timeline of the discovery and description of the dopaminergic neurons in the ventral mesencephalon, it becomes evident that the notion of ‘the’ dopaminergic neuron in this region must be abandoned. Instead we must acknowledge that dopaminergic neurons in the ventral mesencephalon are highly diverse based on their localization, molecular identity, connectome and behavioral relevance. It has long been known that only a specific subtype of dopaminergic neurons succumb to cell death in PD. The precise knowledge of the biology, function and molecular identity of this vulnerable dopaminergic subtype will help us to understand the molecular consequences of harmful genetic and/or environmental events that lead to the outbreak of the disease. Therefore, precisely defining this dopaminergic subtype offers a great potential for new disease‐modifying interventions or refining existing ones, which is the ultimate deliverable of past and ongoing research in this field.
